# Granzyme B is elevated in autoimmune blistering diseases and cleaves key anchoring proteins of the dermal-epidermal junction

**DOI:** 10.1038/s41598-018-28070-0

**Published:** 2018-06-26

**Authors:** Valerio Russo, Theo Klein, Darielle J. Lim, Nestor Solis, Yoan Machado, Sho Hiroyasu, Layla Nabai, Yue Shen, Matthew R. Zeglinski, Hongyan Zhao, Cameron P. Oram, Peter A. Lennox, Nancy Van Laeken, Nick J. Carr, Richard I. Crawford, Claus-Werner Franzke, Christopher M. Overall, David J. Granville

**Affiliations:** 1 International Collaboration On Repair Discoveries (ICORD) Research Centre, Vancouver, BC V5Z 1M9 Canada; 2 Department of Pathology and Laboratory Medicine, University of British Columbia, Vancouver, BC V6T 2B5 Canada; 3 BC Professional Firefighters’ Burn and Wound Healing Research Laboratory, Vancouver, BC V5Z 1M9 Canada; 4 Centre for Blood Research, University of British Columbia, Vancouver, BC V6T 1Z3 Canada; 5 Department of Oral Biological and Medical Sciences, Faculty of Dentistry, University of British Columbia, Vancouver, BC V6T 1Z3 Canada; 6 Department of Surgery, University of British Columbia, Vancouver, BC V5Z 1M9 Canada; 7 Department of Dermatology and Skin Science, University of British Columbia, Vancouver, BC V5Z 4E8 Canada; 8 Department of Dermatology, Medical Center and Faculty of Medicine - University of Freiburg, 79104 Freiburg, Germany

## Abstract

In healthy skin, epidermis and dermis are anchored together at the dermal-epidermal junction (DEJ), a specialized basement membrane pivotal for skin integrity and function. However, increased inflammation in the DEJ is associated with the disruption and separation of this junction and sub-epidermal blistering. Granzyme B (GzmB) is a serine protease secreted by immune cells. Dysregulated inflammation may lead to increased GzmB accumulation and proteolysis in the extracellular milieu. Although elevated GzmB is observed at the level of the DEJ in inflammatory and blistering skin conditions, the present study is the first to explore GzmB in the context of DEJ degradation in autoimmune sub-epidermal blistering. In the present study, GzmB induced separation of the DEJ in healthy human skin. Subsequently, α6/β4 integrin, collagen VII, and collagen XVII were identified as extracellular substrates for GzmB through western blot, and specific cleavage sites were identified by mass spectrometry. In human bullous pemphigoid, dermatitis herpetiformis, and epidermolysis bullosa acquisita, GzmB was elevated at the DEJ when compared to healthy samples, while α6/β4 integrin, collagen VII, and collagen XVII were reduced or absent in the area of blistering. In summary, our results suggest that regardless of the initial causation of sub-epidermal blistering, GzmB activity is a common final pathway that could be amenable to a single targeted treatment approach.

## Introduction

Blistering is a hallmark of many dermatological conditions, and can manifest itself with varying degrees of severity, but is typically characterized by erosions or fluid filled elevations from the skin surface caused by disruption of the cell to cell attachment in different layers of the epidermis, or detachment of the epidermis from dermis. Due to the critical role that skin plays as a barrier in regulating fluid/electrolyte retention, thermoregulation, and protection against infection, depending on the size and severity of blistering, such functions can be compromised and potentially fatal^1^. Based on the etiology, these dermatoses are generally classified in four major groups: (a) antibody-mediated, (b) cutaneous adverse drug reactions, (c) congenital conditions, and (d) blistering caused by external insults such as burns, friction, sunlight, insect bites, and chemical weapons. With respect to autoimmune skin blistering diseases, auto-antibodies are produced against structural or adhesive molecules of the skin and based on the location of the specific auto-antigens and level of the blister formation, these diseases are further classified into intra-epidermal and sub-epidermal blistering diseases. In sub-epidermal blistering dermatoses such as bullous pemphigoid, dermatitis herpetiformis and epidermolysis bullosa acquisita (EBA), auto-antibodies targeting components of the dermal-epidermal junction (DEJ) lead to the disruption of this basement membrane and consequent detachment of the epidermis^2^.

Granzyme B (GzmB) is a serine protease widely known for its pro-apoptotic role in cytotoxic T lymphocyte (CTL)- and natural killer (NK) cell-mediated killing of target cells whereby the pore-forming protein perforin is secreted along with GzmB and facilitates its entry into target cells^3^. However, in recent years it has become clear that GzmB accumulation in the extracellular space can contribute to other pathological processes. Indeed, the directed secretion of GzmB from the effector cell towards the target cell is not efficient, resulting in leakage into the extracellular milieu^4^. Furthermore, it is now recognized that other immune and non-immune cell types, that do not express perforin and/or form immunological synapses such as plasmacytoid dendritic cells, B cells, mast cells, and keratinocytes may also express and secrete GzmB under certain conditions (reviewed in^5^). As such, the extracellular function of GzmB has received much attention in recent years as its role in the onset of several inflammatory conditions continues to be revealed. GzmB is capable of cleaving cell receptors, cellular adhesion proteins, cytokines and important extracellular matrix (ECM) proteins, thus affecting tissue structure and function^6–8^. Of particular relevance to autoimmune skin blistering, GzmB accumulation at the DEJ has been reported by previous studies in bullous diseases and cutaneous adverse drug reactions^9–11^. However, with respect to mechanism of action in skin diseases, GzmB has been viewed almost exclusively in the context of CTL/NK-mediated keratinocyte apoptosis^12–15^ while the recently recognized role of extracellular GzmB proteolysis^5,8,16^ has not been considered.

As GzmB accumulates on both sides of the DEJ, including the dermis which is devoid of keratinocytes, and given the established potential of this enzyme to cleave multiple ECM proteins, we hypothesized that GzmB compromises DEJ integrity and function through cleavage of key basement membrane components α6/β4 integrin, collagen VII, and collagen XVII, thus directly contributing to epidermal detachment and blistering through extracellular mechanisms. Using *in vitro* cleavage assays and amino-terminal oriented mass spectrometry (ATOMS)^17,18^ on purified proteins and intact human skin, we provide evidence that GzmB cleaves important proteins of the DEJ and induces epidermal detachment of healthy skin. This work demonstrates for the first time a role for extracellular GzmB in the blistering process that goes beyond a cytotoxic effect on basal keratinocytes.

## Results

### GzmB accumulates at the level of the DEJ in bullous pemphigoid, dermatitis herpetiformis, and EBA

**Figure 1:**
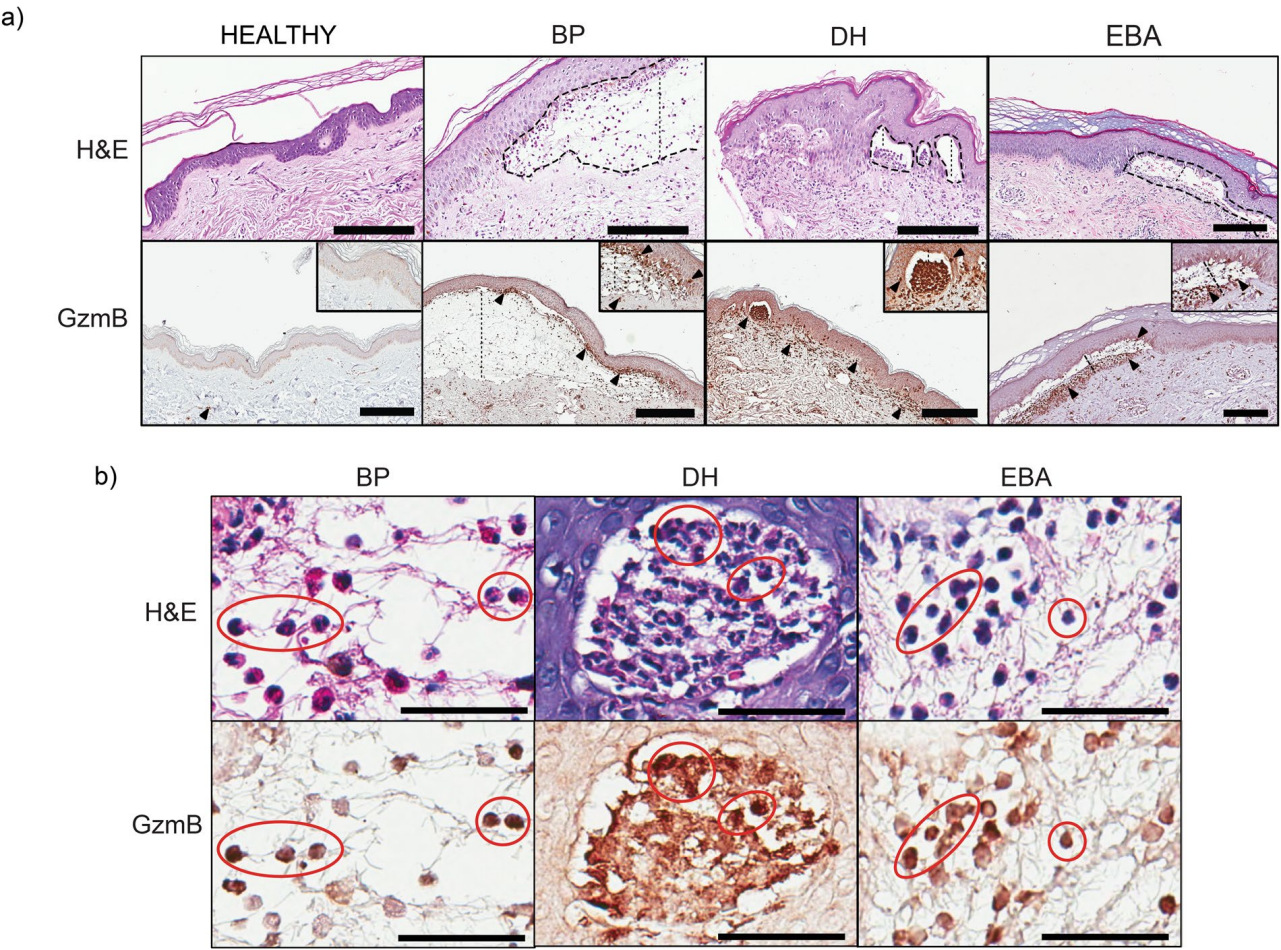
GzmB levels are elevated in the DEJ of sub-epidermal blistering diseases. (**a**) In the upper row, representative images of H&E staining of healthy skin, bullous pemphigoid (BP), dermatitis herpetiformis (DH), and epidermolysis bullosa acquisita (EBA). Dotted lines delineate blisters. In the lower row, GzmB immunostaining of healthy, bullous pemphigoid (BP), dermatitis herpetiformis (DH), and epidermolysis bullosa acquisita (EBA) biopsies. Abundant GzmB is observed at the level of the dermal-epidermal junction in diseased skin particularly in areas of epidermal separation (black arrowheads). Dotted lines indicate separation between the epidermis and the dermis. Scale bars represent 200 μm. (**b**) In the upper row, representative images of H&E staining of, bullous pemphigoid (BP), dermatitis herpetiformis (DH), and epidermolysis bullosa acquisita (EBA). In lower row, GzmB staining for the same tissue sections. In all conditions studied GzmB co-localizes with neutrophils (red circles). Scale bars represent 40 μm.

Immunohistochemistry of patient skin samples from bullous pemphigoid, dermatitis herpetiformis, and EBA indicated that GzmB accumulated at the DEJ. Specifically, H&E staining of bullous pemphigoid revealed sub-epidermal blistering with dense inflammatory infiltrate consisting predominantly of eosinophils and neutrophils (Fig. 1a,b). Intense GzmB staining was observed at the level of the DEJ in most neutrophils but not in eosinophils as expected, both within the blister and immediately below the detached epidermal layer (Fig. 1b). Dermatitis herpetiformis was characterized by pathognomonic sub-epidermal clefts and papillary abscesses, consisting mostly of neutrophils and a few eosinophils, at the tips of dermal papillae (Fig. 1a,b). These papillary abscesses were heavily stained for GzmB suggesting neutrophil and lymphocyte involvement in its secretion (Fig. 1b). GzmB presence was predominantly observed in the upper papillary dermis adjacent to the DEJ, but positive cells could also be detected embedded within the epidermal layer. EBA skin displayed epidermal detachment with abundant immune infiltrate, mostly composed of neutrophils and lymphocytes, in the interstitial space between the separated epidermis and the dermis (Fig. 1a,b). Similar to what observed in bullous pemphigoid and dermatitis herpetiformis, GzmB was found predominantly in neutrophils (Fig. 1b).

### GzmB cleaves α6 and β4 integrins *in vitro* in domains pivotal for their function

**Figure 2:**
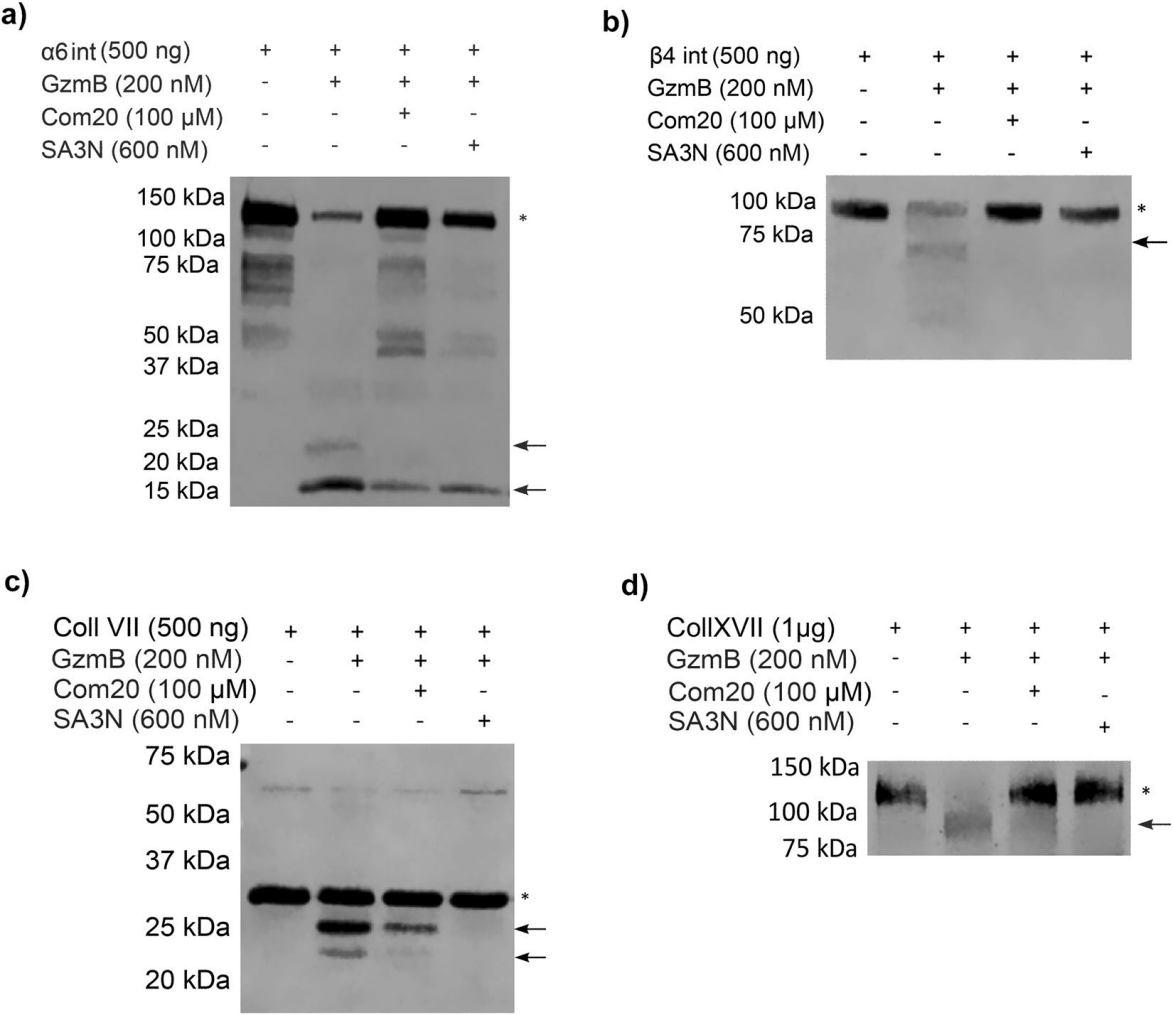
GzmB Cleavage assays on dermal-epidermal junction proteins. (**a**) 4–20% SDS-PAGE western blot of GzmB-mediated cleavage of α6 integrin (α6 int) sub-unit with and without inhibitors serpin A3N (SA3N) and compound 20 (Com20). Black arrows indicate cleavage fragments and * indicates full-length proteins. At a concentration of 200 nM GzmB produces cleavage bands, and this cleavage is prevented by GzmB inhibitors. Full-length blot is presented in Supplemental Fig. S5. (**b**) 4–20% SDS-PAGE western blot of GzmB-mediated cleavage of β4 integrin sub-unit (β4 int) with and without inhibitors SA3N and Com20. Black arrows indicate cleavage fragments and * indicates full-length proteins. At a concentration of 200 nM GzmB produces cleavage bands, but its inhibition prevents the appearance of these bands. Full-length blot is presented in Supplemental Fig. S6. (**c**) 10% SDS-PAGE western blot of GzmB-mediated cleavage of collagen VII (coll VII) with and without inhibitors SA3N and Com20. Black arrows indicate cleavage fragments and * indicates full-length proteins. GzmB produces cleavage bands, and this cleavage is reduced by the addition of Com20 and abolished by SA3N. Full-length blot is presented in Supplemental Fig. S7. (**d**) 8% SDS-PAGE western blot of GzmB-mediated cleavage of collagen XVII (coll XVII) with and without inhibitors SA3N and Com20. Black arrow indicates cleavage fragment and * indicates full-length protein. GzmB produces a cleavage band, and this cleavage is abolished by the addition of Com20 or SA3N. Full-length blot is presented in Supplemental Fig. S8.

**Figure 3:**
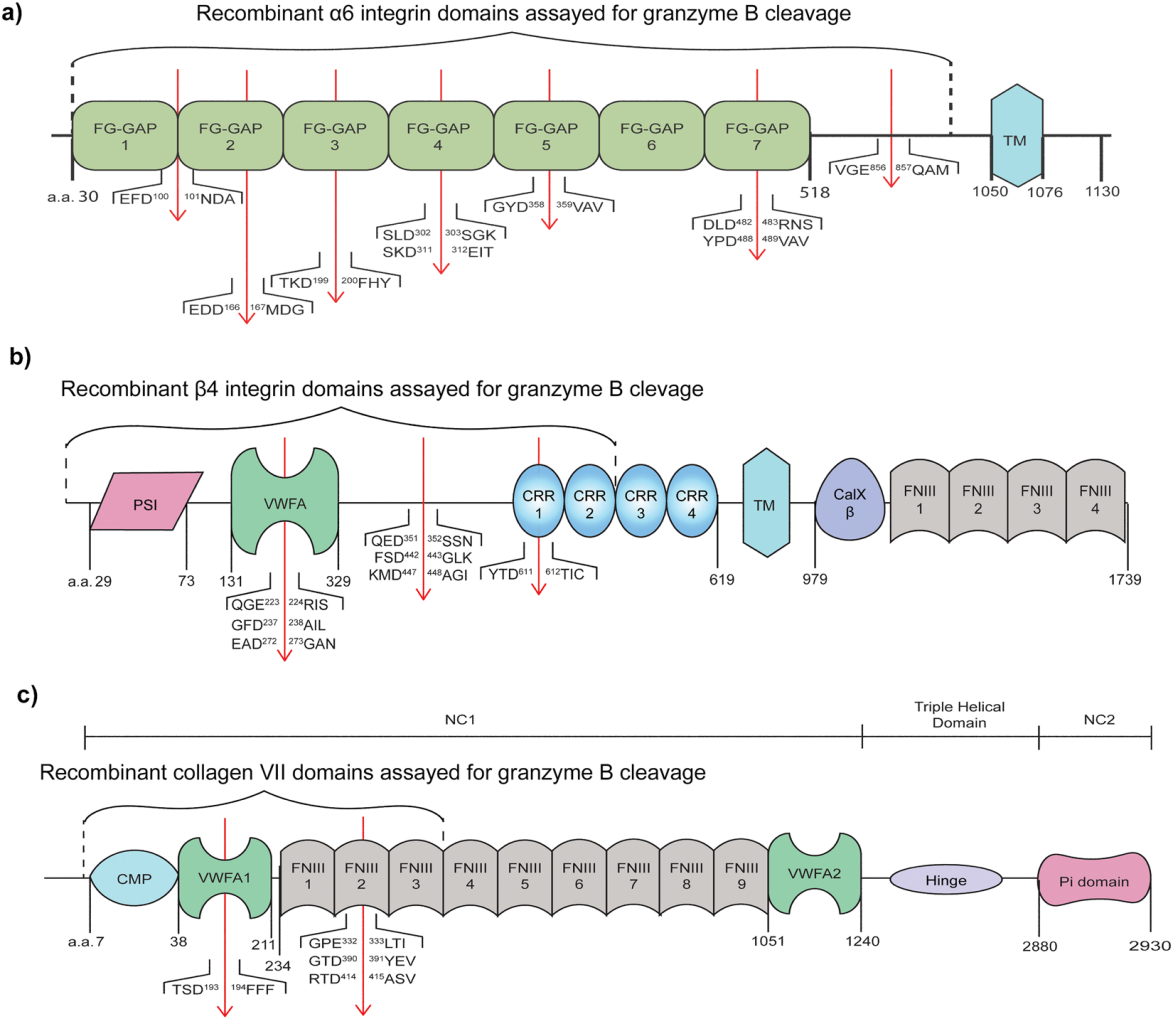
Mass spectrometry analysis on GzmB-cleaved DEJ substrates. (**a**) Extracellular domain schematics for α6 integrin. GzmB mediated cleavage sites identified proteomically by ATOMs (red arrows) fall within the ligand-binding domains in the β-propeller region. GzmB, granzyme B; TM, transmembrane helix. (**b**) Extracellular domain schematics for β4 integrin. GzmB mediated cleavage sites identified proteomically by ATOMS (red arrows) falls within ligand-binding domains in the specificity-determining loop. (**c**) Extracellular domain schematics for collagen VII. GzmB mediated cleavage sites identified proteomically by ATOMS (red arrows) falls in the von Willebrand factor A and fibronectin type III-2 domains, which mediate collagen VII attachment to other dermal-epidermal junction components, such as laminins and collagen IV. TM, transmembrane helix; PSI, plexin-semaphorin-integrin; VWFA, von Willebrand factor A; CRR, cysteine-rich region; FNIII, fibronectin-like III domain; NC, non collagenous region; CMP, cartilage matrix protein; VWFA1/2, von Willebrand factor A 1/2; Pi, protein inhibitor.

Once we ascertained GzmB presence at the level of the DEJ in diseased skin, we hypothesized that GzmB mediates cleavage of α6/β4 integrin, collagen VII, and collagen XVII, which are important components of the basement membrane critical for DEJ function. Both α6 and β4 integrin sub-units were cleaved by GzmB (Fig. 2a,b); α6 integrin was detected at 150 kDa with fragments at ~20, 25, and 37 kDa, whereas cleavage of β4 integrin ectodomain (100 kDa) yielded an evident fragment at ~65 kDa and a weaker band at 50 kDa. To confirm that cleavage of these DEJ components was indeed mediated by GzmB, compound 20, a GzmB-specific competitive inhibitor, and serpin A3N, an irreversible serine protease inhibitor, were included in the cleavage assay. Both inhibitors prevented the cleavage of α6 and β4 integrin sub-units at 100 μM and 600 nM respectively (Fig. 2a,b). Furthermore, mass spectrometry by ATOMS was used to identify cleavage sites on these proteins to assess whether GzmB-mediated cleavage of α6 and β4 integrins could impair DEJ function. We focussed on the extracellular domains of α6 and β4 integrins as this protein region is more likely to be exposed to GzmB. α6 integrin was cleaved by GzmB at Asp100, Asp166, Asp199, Asp302, Asp311, Asp358, Asp482, and Asp488 in the FG-GAP repeats 2, 3, 4, 5, and 7 within the extracellular β-propeller domain (Fig. 3a and Supplemental Fig. S1), a cleavage site at Glu856 was also detected. Cleavage of the β4 integrin sub-unit fell within the Von Willebrand factor A domain at Glu223, Asp237 and Asp272, as well as within the Cysteine Rich Region 1 at Asp611, and within the linker region between these two domains at Asp351, Asp442 and Asp447 (Fig. 3b and Supplemental Fig. S2).

### GzmB cleaves collagen VII in ligand binding regions

Western blot was used to assess cleavage of collagen VII domain a.a 199–482 by GzmB. This fragment is part of the non collagenous region 1 (NC1), which is pivotal for collagen VII interactions with other proteins of the ECM^19,20^. Untreated collagen VII fragment was detected at ~30 kDa, and its cleavage by GzmB yielded bands at ~20 and 25 kDa (Fig. 2c). On the other hand, collagen I, the most common collagen in the human body, was not cleaved by GzmB (Supplemental Fig. S3). Inhibition of collagen VII cleavage with serpin A3N prevented the appearance of both ~20 and 25 kDa bands, whereas compound 20 inhibition was incomplete and a cleavage band could still be detected at ~25 kDa (Fig. 2c). As for the location of cleavage, the NC1 fragment of collagen VII we tested was cleaved by GzmB in the Von Willebrand factor A domain at Asp193, in the fibronectin-like domain III-2 at Glu332 and Asp390, and within fibronectin-like domain III-3 at Asp414 (Fig. 3c and Supplemental Fig. S4).

### Collagen XVII is a substrate for GzmB cleavage

As a crucial component of the hemidesmosomes, collagen XVII plays a critical role in bridging the intracellular and the extracellular structural elements involved in epidermal adhesion^21^. Treatment of collagen XVII ectodomain (a.a 490–1497) with GzmB resulted in cleavage of this region, and in the appearance of a cleavage band at ~95 kDa (full length protein ~120 kDa). Pre-incubation of GzmB with both compound 20 and serpin A3N prevented NC16 cleavage (Fig. 2d). ATOMS was attempted on collagen XVII using 1 or 2 µg of protein. GzmB-digested and undigested forms of collagen XVII were compared by labeling exposed N-termini with heavy (^13^CD_2_O formaldehyde) and light (^12^CH_2_O formaldehyde) dimethylated tags respectively. However, detection of N-terminal peptides was confounded due to ion suppression by more intense ions from other contaminating proteins in the sample. Furthermore, agglutination of collagen XVII was observed after dimethylation, which likely decreased the efficiency of trypsin digestion. For these reasons, we were unable to obtain cleavage site data for this protein.

### α6/β4 integrin, collagen VII and collagen XVII lining at the DEJ are disrupted in diseased skin

**Figure 4:**
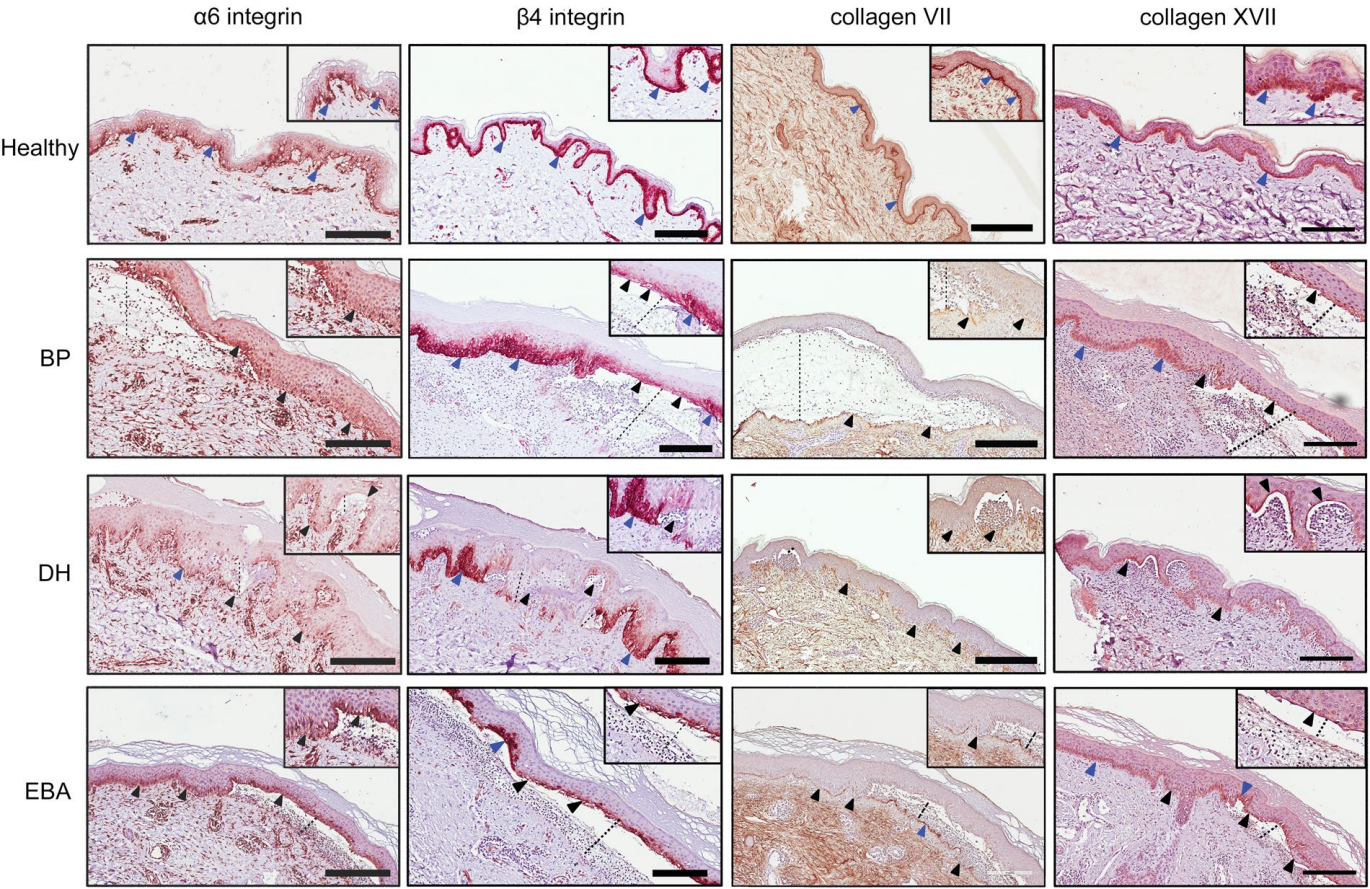
Immunohistochemistry of DEJ components in healthy and diseased skin biopsies. α6 and β4 integrin, collagen VII, and collagen XVII immunostaining of healthy, bullous pemphigoid (BP), dermatitis herpetiformis (DH), and epidermolysis bullosa acquisita (EBA) biopsies. Blue arrowheads indicate intact protein in areas of dermo-epidermal adhesion, black arrowheads indicate weak or absent staining. Dotted lines indicate separation between the epidermis and the dermis. Scale bars represent 200 μm. **α6 integrin** is fragmented and scattered throughout the dermis in diseased skin, whereas in healthy biopsies this protein lines the DEJ. **β4 integrin** appears to be crucial for adhesion: in the bullous pemphigoid sample, a flap of dermis in the lower right corner is attached to the epidermis and shows strong β4 staining; this area is flanked by separated epidermis with faint β4 integrin staining. Scale bars represent 200 μm. **Collagen VII** lining is intact (blue arrowheads) in healthy skin, but weak or absent immunoreactivity was observed in diseased samples (black arrowheads). **Collagen XVII** lining is intact (blue arrowheads) in healthy skin, but weak or absent immunoreactivity was observed in diseased samples (black arrowheads).

Following western blot and ATOMS analyses indicating that both α6 and β4 integrin sub-units, collagen VII and collagen XVII are substrates for GzmB, we sought to assess their integrity in bullous pemphigoid, dermatitis herpetiformis, and EBA skin samples. Staining for α6 integrin in normal skin was mainly localized perivascularly in the dermis and as a continuous line at the DEJ. When diseased skin samples were investigated, the pattern of α6 integrin localization was similar for all conditions, exhibiting scattered staining throughout the entire sections, which could be indicative of protein fragmentation (Fig. 4). At the DEJ, staining was absent, weak, or disorganized both in areas of epidermal detachment and in sections where the epidermis was still attached to the dermis (Fig. 4). As for integrin β4, a strong well localized staining delineated the DEJ in healthy skin, as well as in bullous pemphigoid and dermatitis herpetiformis in areas where the epidermis was anchored to the dermis (Fig. 4). However, integrin β4 staining was completely absent or faint in areas of epidermal separation in all conditions studied (Fig. 4).

Mooney *et al*. have previously shown fragmented collagen VII in the DEJ of patients with discoid lupus erythematosus^22^. While normal skin displayed a continuous, strong staining for collagen VII lining the interface between epidermis and dermis (Fig. 4), in bullous pemphigoid and dermatitis herpetiformis collagen VII staining was weak or absent. Weak staining in both diseases was localized on the dermal side of a blister or papillary abscess, consistent with a separation of the fibrillar zone of the DEJ from the lamina densa above it due to cleavage of collagen VII in the NC1 (Fig. 4). In dermatitis herpetiformis, collagen VII staining presented a peculiar pattern, with short stained sections perpendicular to the epidermis rather than parallel to it. Collagen VII staining of EBA samples showed an intermittent pattern, with DEJ segments presenting weak staining alternated by areas with a stronger staining (Fig. 4).

Finally, collagen XVII staining pattern for all conditions was similar to what was observed for the other substrates. A clear, uninterrupted line of staining was detected in healthy skin, whereas in diseased skin weak staining was observed in areas of reduced DEJ integrity and was mostly absent in the sections of skin where the epidermis had detached (Fig. 4).

### Incubation with GzmB results in epidermal separation in healthy human skin and is inhibited by Compound 20

**Figure 5:**
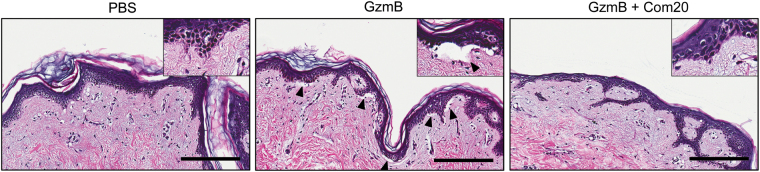
GzmB induces DEJ separation. H&E staining of healthy skin incubated for 12 h at 37 °C in PBS, 200 nM GzmB, or 200 nM GzmB previously inactivated with 100 μM Compound 20 (Com20). Clefts between the epidermis and the dermis (black arrowheads) were observed in GzmB-treated samples but were absent in PBS control and in the sample where GzmB was inhibited by Com20. Scale bars represent 200 μm.

As GzmB is elevated at the DEJ of bullous pemphigoid, dermatitis herpetiformis, and EBA, and capable of cleaving key junctional proteins, the direct impact of GzmB proteolysis on DEJ integrity in freshly-isolated human skin was assessed. A ~1 mm × 4 mm strip of healthy skin comprising epidermis and dermis was immersed and incubated for 12 h at 37 °C in a 200 nM solution of GzmB. Upon incubation, the skin was viable but H&E staining revealed the appearance of clefts between the epidermis and the dermis in the GzmB-treated sample that were mostly absent in the PBS control (Fig. 5 and Supplemental Fig. S9). Inactivation of GzmB with the GzmB-specific inhibitor Compound 20 prevented the formation of DEJ clefts, revealing a tissue morphology similar to PBS control (Fig. 5).

## Discussion

It is now widely acknowledged in the literature that extracellular GzmB exerts a pathogenic, perforin-independent, role in conditions associated with dysregulated and/or chronic inflammation and impaired tissue repair due to ECM cleavage^7,16,23–25^. In the present study, we used western blot and ATOMS to show for the first time that α6/β4 integrin and collagen VII, which are key components of the DEJ, are cleaved by GzmB in key regions for their anchoring functions. We also demonstrated that in diseased human skin biopsies of bullous pemphigoid, dermatitis herpetiformis and EBA, sub-epidermal blisters display elevated levels of GzmB at the DEJ accompanied by degradation of the newly discovered GzmB substrates α6/β4 integrin, collagen VII, and collagen XVII. Supporting the pathological relevance of GzmB activity, we reported partial detachment of the epidermis from the dermis in healthy human skin exposed to a physiologically relevant concentration of GzmB^26,27^, and showed that this separation can be prevented by a GzmB-specific inhibitor. These data suggest that, in addition to previously studied proteases^28^, extracellular GzmB could also contribute to epidermal separation in autoimmune sub-epidermal blistering pathologies.

Evidence for the importance of α6/β4 integrin comes from both animal models^29^ and from severe, often lethal, human blistering diseases. Mutations and deficiencies of the integrin complex involving α6/β4, as well as production of anti-α6/β4 integrin auto-antibodies^30,31^, contribute to lethal phenotypes characterized by widespread muco-cutaneous blistering. Several studies have identified mutations in the genes coding for α6/β4 integrin^32–34^ or absence at the protein level of one of its sub-units^35^, in numerous subtypes of epidermolysis bullosa. In particular, Phillips *et al*. speculated that *in situ* proteolytic cleavage of the epitopes by an as yet unidentified protease, might be responsible for the loss of β4 sub-unit immunoreactivity in patients with junctional epidermolysis bullosa^36^. In this study we show that GzmB cleaves both α6 and β4 integrin sub-units at several sites in their ligand-binding extracellular domains^37–40^, possibly compromising the adhesive properties of this molecule. The importance of these extracellular regions is particularly evident for integrin β4 as collectively, most of the missense mutations and the amino acid deletions described in lethal junctional epidermolysis bullosa were located in its extracellular domain^32,41–43^, while missense or splice mutations associated with the non-lethal form were frequently located in the cytoplasmic portion^41,44,45^. Interestingly, Nakano *et al*. identified the lethal mutation p.D131Y/p.G273D, which may abolish important ligand binding sites of integrin β4 as it falls within a highly conserved region^41^. Since one of the GzmB cleavage sites we have identified is at Asp272, this is suggestive that GzmB-mediated cleavage of α6/β4 integrin could likewise severely affect the adhesive properties of this molecule.

As the main component of the papillary dermis, another protein fundamental for the integrity of the DEJ is collagen VII. Among collagen VII domains, fibronectin-like regions are pivotal for the interaction with several ECM components. Studies with a recombinant version of the NC1 region revealed strong binding affinity of fibronectin-like domains with collagen I, collagen IV, laminin 332 and fibronectin^19,20^, which allows anchorage of the papillary dermis to the lamina densa. Mutations of collagen VII or production of auto-antibodies against this region result in severe disruption of the DEJ, causing dystrophic epidermolysis bullosa and EBA respectively^46,47^. These conditions are characterized by extensive epidermal detachment and formation of blisters. We show herein GzmB-mediated cleavage of collagen VII in the fibronectin-like III-2 domain, as well as in the von Willebrand factor A domain, and inhibition of this cleavage by the GzmB inhibitors serpin A3N and compound 20^48^. Moreover, GzmB-mediated cleavage of collagen XVII was also observed. Collagen XVII is another important component of the hemidesmosome, whose interaction with α6/β4 integrin is required for the assembly of protein complexes that anchor basal keratinocytes to the lamina lucida^44^. Taken together, these observations suggest that GzmB can disrupt the basal keratinocyte/lamina lucida connection through cleavage of α6/β4 integrin and collagen XVII, and lamina densa/fibrillar zone adhesion through cleavage of collagen VII.

GzmB accumulation at the DEJ is observed in bullous dermatoses such bullous pemphigoid^9^ and dermatitis herpetiformis^10^, as well as in cutaneous adverse drug reactions including lichenoid drug eruption^49^, SJS/TEN and generalized bullous fixed drug eruption^11^. However, as the newly discovered, non-apoptotic roles for GzmB were not established at the time, none of the aforementioned studies considered extracellular GzmB-mediated proteolysis as a mechanism for DEJ disruption. Rather, GzmB was proposed to contribute to CD8^+^ T-cell- and NK-mediated, keratinocyte and melanocyte^50^ apoptosis in a perforin-dependent manner. While mostly absent in normal skin, GzmB is present at the DEJ in the sub-epidermal blistering conditions bullous pemphigoid and dermatitis herpetiformis in agreement with previous studies^9–11^, and we have shown for the first time accumulation of this protease at the DEJ in EBA. It has long been hypothesized that anti-DEJ auto-antibody-triggered sub-epidermal blister formation is mediated by proteases secreted by infiltrating inflammatory cells^51^. Indeed, auto-antibody-mediated immune cell recruitment to the DEJ is a mandatory condition for dermo-epidermal separation as it dictates the localized and concentrated degranulation of proteinases^52^. Confirming this mechanism, auto-antibodies contained in the serum of patients with bullous pemphigoid^53^, EBA^54^, and pemphigoid gestationis^55^ promote leukocyte recruitment to the DEJ resulting in its separation. We suggest that GzmB could be an important contributor to this DEJ separation in autoimmune conditions as the DEJ substrates we have identified are degraded proximal to the area in which blistering is occurring in these diseases. Although *in vivo* studies will be necessary to fully elucidate GzmB contribution to autoimmune skin blistering, further evidence for a role of GzmB in this process derives from our observation that incubation of healthy human skin with a physiologically relevant concentration of this protease results in clefts formation at the DEJ. One limitation of this model is that, unlike in diseased skin, staining for α6/β4, collagen VII, and collagen XVII remained detectable in areas of epidermal separation (data not shown). There are several explanations for this result. Firstly, in this model the entire skin section is bathed in a solution containing cationic GzmB, which is attracted to, and sequestered by negatively charged proteoglycans rather than concentrating at the DEJ, whereas in sub-epidermal blistering conditions, GzmB is directed specifically to the basement membrane by auto-antibodies. Thus, it is possible that in areas of mild epidermal separation observed in our *ex vivo* model the antibodies we used for IHC could still recognize their epitopes, while the lack of staining in diseased human samples characterized by complete epidermal detachment is a consequence of more severe protein degradation due to a long-term and DEJ-localized exposure to GzmB. Another explanation is that given this is a static environment, cleavage fragments likely remain in the DEJ and are subsequently detected by immunohistochemistry. Despite these limitations, this model shows a causative relation between GzmB exposure and epidermal separation, although not as widespread and dramatic as observed *in vivo*, providing proof of concept that GzmB alone can dramatically affect DEJ anchoring function.

The pathological role of extracellular GzmB in autoimmune diseases might not be limited to the physical disruption of important substrates. Growing evidence suggest that antigenic, GzmB-generated peptide fragments are part of a feed-forward loop that sustains the propagation of several autoimmune diseases (reviewed in^56^). Studies suggest that GzmB is instrumental in auto-antigen generation in certain autoimmune conditions^57–59^. In our study, GzmB cleaves α6/β4 integrin and collagen VII in epitope regions recognized by auto-antibodies present in the sera of patients with certain pemphigoid diseases, and EBA respectively. In oral pemphigoid, one of the identified auto-epitopes is represented by the peptide a.a. 292–305 of α6 integrin^60^. *In vitro*, GzmB cleaves α6 integrin at residues Asp199 and Asp302, thus we speculate that GzmB could potentially generate this antigenic fragment *in vivo*, establishing a cycle of sustained immune response and further generation of antigenic fragments.

As mentioned above, we also observed GzmB-mediated cleavage of the ectodomain of collagen XVII. The production of collagen XVII auto-antibodies results in bullous pemphigoid^61,62^ whereas mutations in the ectodomain of this protein have been associated with certain forms of junctional epidermolysis bullosa^63,64^. Although we were not able to identify the specific GzmB cleavage sites in the NC16 region of collagen XVII, this domain is the immunodominant region in bullous pemphigoid and its recombinant forms are used for detecting specific auto-antibodies in approximately 85% of patients affected by this condition^65,66^.

Finally, several groups have demonstrated that in EBA, T and B cells target identical regions of the NC1 domain of collagen VII^67,68^. In particular, Lapiere *et al*. incubated different fragments of collagen VII with sera from 19 EBA patients, and observed that 16 sera strongly reacted with the fusion protein composed of the fibronectin-like III domains 1 to 4^69^. The NC1 domain of collagen VII also mediates Fc-dependent neutrophil activation and induction of dermo-epidermal separation^54^.

Given the presence of GzmB cleavage sites in the immunodominant regions of α6/β4 integrin, collagen XVII, and collagen VII, this protease may contribute to the generation of antigenic peptides either directly, or by revealing a cryptic epitope due to a change in substrate structure. If confirmed, this hypothesis would have substantial therapeutic relevance as the molecular target of GzmB includes, but is not limited to the antigenic target of the auto-antibodies. Thus, even though the disease-defining antigenic targets are all different, they result in a common final pathway that may be amenable to the single targeted treatment approach of inhibiting GzmB.

In summary, the present study suggests for the first time that GzmB mighty directly contribute to sub-epidermal autoimmune blistering via extracellular proteolysis-mediated DEJ impairment, thus playing a role in this pathological process that goes beyond cytotoxicity. Inhibition of GzmB represents a novel therapeutic approach for the treatment and prevention of autoimmune sub-epidermal blistering.

## Materials and Methods

All human studies were approved by the University of British Columbia Review Ethic Board (H15-03345). Waiver of consent was obtained as per Tri-Council Policy Statement article 12.3B.

### Immunohistochemistry and Histology on Diseased Skin

Paraffin-embedded skin samples from patients affected by bullous pemphigoid (N = 3), dermatitis herpetiformis (N = 3), and inflammatory EBA (N = 3) were sectioned (5 μm) for immunohistochemical analysis of GzmB (Cat # ab4059, ABCAM) and collagen VII (Cat # ab93350, ABCAM, Toronto, ON) using 3,3′-Diaminobenzidine for visualization, as well as α6 (Cat # ab181551, ABCAM) and β4 (Cat # ab182120, ABCAM) integrins, and collagen XVII (NC16a-3^70^) visualized through Novared®. Healthy skin obtained from patients undergoing elective abdominoplasty was used as a control. In order to observe cellular infiltrate and tissue architecture, H&E staining was also performed using established methods. Following image acquisition, these H&E stained sections were de-stained by serial incubation in xylene (2 min), 100% EtOH (2 min), 95% EtOH (2 min), water (10 min), and acidic alcohol solution (10 min). In order to detect GzmB-producing cells, de-stained H&E sections were probed for GzmB (Cat # ab4059, ABCAM). All slides were scanned using a Aperio CS2 slide scanner (Leica, Concord, ON).

### DEJ Proteins Cleavage Assay

The recombinant human integrin α6/β4 (α6 a.a. 24–878, β4 a.a. 28–710, Cat # 5497-A6-050, R&D Systems, Minneapolis, MN), collagen VII (199–482 a.a., Cat # 2018615, MyBiosource, San Diego, CA), collagen XVII ectodomain (490–1497 a.a., generous gift from Dr. Claus-Werner Franzke), and collagen I (Cat # NBP1-97266, Novus Biologicals, Littleton, CO) were incubated for 24 h at 37 °C in 200 nM purified human GzmB (EmeraldBio, Bainbridge Island, WA). For inhibition studies, prior to the addition of substrates to the reaction, GzmB was incubated in the presence of 600 nm serpin A3N (generous gift from Dr. Chris R. Bleackley, University of Alberta, Edmonton, AB, Canada) or 100 μM of the small molecule inhibitor compound 20 (courtesy of the Centre for Drug Research and Development, Vancouver, BC) for 1 h at 37 °C. After the 24 h incubation, proteins were denatured, separated on a 4–20% (for integrin α6/β4), 10% (for collagen VII), 8–10% (for collagen XVII), or 7.5% (for collagen I) SDS-polyacrylamide gel. Cleavage was detected by western blot using anti-human integrin α6 sub-unit (Cat # ab181551, ABCAM), anti-human integrin β4 sub-unit (Cat # MAB4060, R&D Systems, Minneapolis, MN), anti-collagen VII (Cat # ab93350, ABCAM), NC16A antibody directed against the NC16A domain of human collagen XVII^71^ and imaged using a Li-Cor Odissey FC (Li-Cor, Lincoln, NE). Coomassie staining was used for the assessment of collagen I cleavage following manufacturer instructions.

### LC-MS/MS analysis

GzmB cleavage sites were identified by ATOMS as described earlier^17,18^. Briefly, GzmB-digested or control substrates were denatured, cysteines reduced with dithiothreitol and alkylated with iodoacetamide. Primary amine groups were dimethylated with formaldehyde and sodium cyanoborohydride before acetone precipitation. Pellets were redissolved in digestion buffer with 1 μg/mL MS-grade trypsin (Thermo Fisher Scientific, Waltham, MA) and desalted using StageTips^72^. Samples were analyzed on an Impact II Q-TOF (Bruker, Billerica, MA) on ReproSilPur 120 C18-AQ 1.9 μm particles (Dr. Maisch) columns and resolved by a gradient of acetonitrile (0.1% v/v formic acid) in water delivered by an easy-nLC system (Thermo Fisher Scientific) preceding data-dependent precursor selection of the top 18 peaks. Spectra were extracted using DataAnalysis 4.3 (Bruker) and searched against a Uniprot human proteome database (downloaded 2016-02-24; 70,472 sequences) using semi-specific ArgC as enzyme specificity, carbamidomethylation (C) and dimethylation (K) as fixed modifications, and deamidation (N,Q), dimethylation (N-term), pyroglutamation (Q) and oxidation (M) as variable modifications. Potential GzmB cleavage sites were defined as semi-specific, N-terminally dimethylated peptides with an acidic residue N-terminal to the identified sequence identified at a peptide expect value <0.01.

### Skin Cleavage Assay

Fresh healthy human skin obtained from patients undergoing elective plastic surgery was transported to the lab and cut into ≈1 mm × 4 mm strips (N = 3). The adipose tissue layer was removed to obtain a strip of dermis and epidermis. Skin strips were then incubated at 37 °C for 12 h in 300 μL of either PBS, 200 nM GzmB, or 200 nM GzmB previously inactivated through 1 h incubation at 37 °C with 100 μM Compound 20. Following incubation, samples were fixed in 10% buffered formalin overnight, paraffin embedded and sectioned for H&E staining using established methods, and TUNEL (cell death) analysis following manufacturer’s instructions (Cat # 11684795910, Sigma). Outermost sections of the samples were used for staining, as GzmB might not penetrate deep into the tissue.

## Electronic supplementary material

Supplemental Information
