# Supplementary material for: Granzyme B is elevated in autoimmune blistering diseases and cleaves key anchoring proteins of the dermal-epidermal junction
Source: Sci Rep. 2018 Jun 26;8:9690. doi: 10.1038/s41598-018-28070-0 (PMC6018769; doi:10.1038/s41598-018-28070-0)
Supplement: Supplementary file 1 — Supplemental Information [file 41598_2018_28070_MOESM1_ESM.pdf]

**Granzyme B is elevated in autoimmune blistering diseases and cleaves key anchoring proteins of the dermal-epidermal junction**

Valerio Russo [1,2,3], Theo Klein [4,5], Darielle J. Lim [1], Nestor Solis [4,5], Yoan Machado [4,5], Sho Hiroyasu [1, 2, 3], Layla Nabai [1,3], Yue Shen [1,2,3], Matthew R. Zeglinski [1,2,3], Hongyan Zhao [1,2,3], Cameron P. Oram [1,2,3], Peter A. Lennox [6], Nancy Van Laeken [6], Nick J. Carr [6], Richard I. Crawford [2,7], Claus-Werner Franzke [8], Christopher M. Overall [4,5], David J. Granville [1,2,3,*]

[1] International Collaboration On Repair Discoveries (ICORD) Research Centre, Vancouver, BC, Canada V5Z 1M9
[2] Department of Pathology and Laboratory Medicine, University of British Columbia, Vancouver, BC, Canada V6T 2B5
[3] BC Professional Firefighters' Burn and Wound Healing Research Laboratory, Vancouver, BC, Canada V5Z 1M9
[4] Centre for Blood Research, University of British Columbia, Vancouver, BC, Canada V6T 1Z3
[5] Department of Oral Biological and Medical Sciences, Faculty of Dentistry, University of British Columbia, Vancouver, BC, Canada V6T 1Z3
[6] Department of Surgery, University of British Columbia, Vancouver, BC, Canada V5Z 1M9
[7] Department of Dermatology and Skin Science, University of British Columbia, Vancouver, BC, Canada V5Z 4E8
[8] Department of Dermatology, Medical Center and Faculty of Medicine - University of Freiburg, 79104 Freiburg, Germany

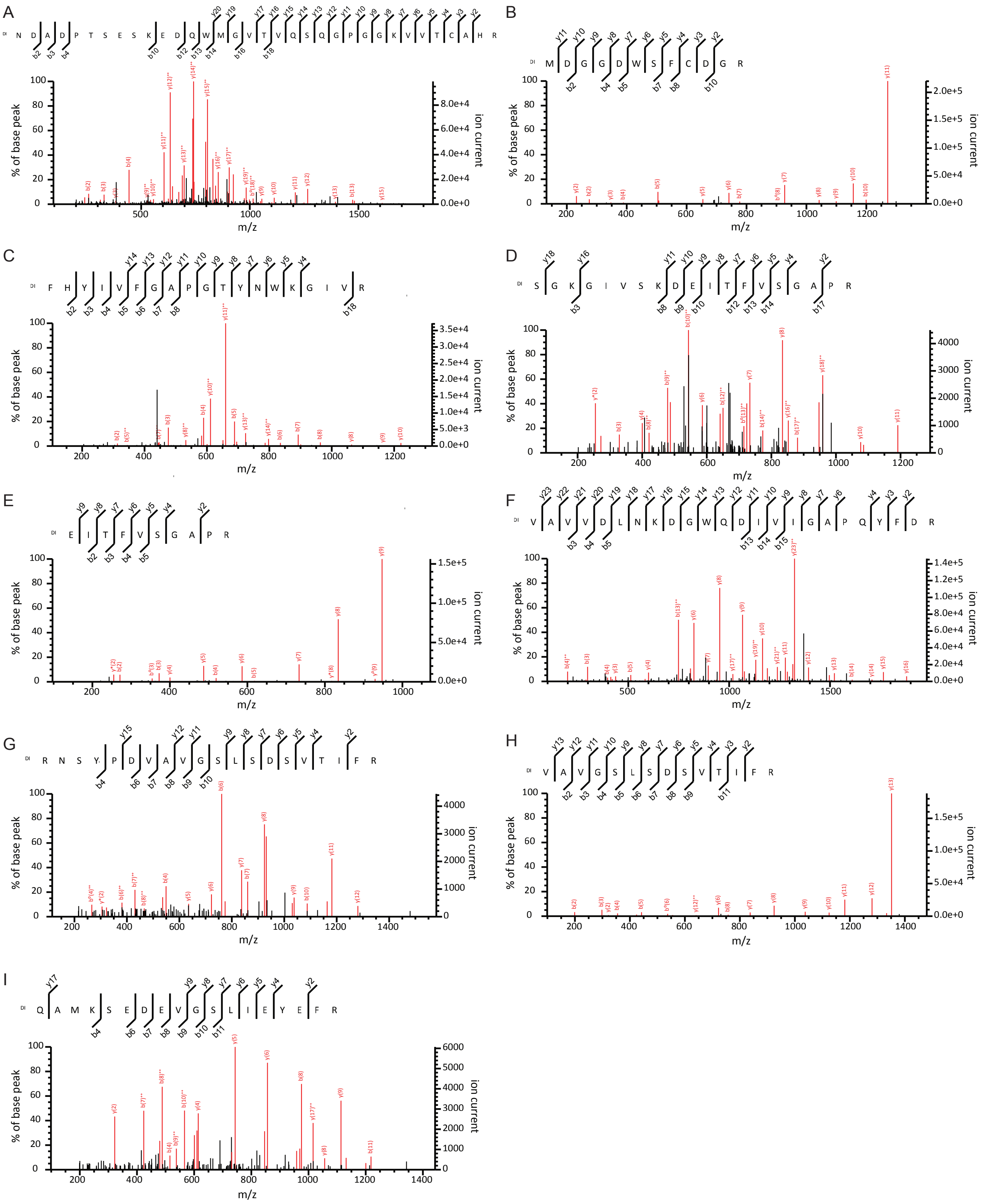


**Supplemental Figure S1:** Annotated MS/MS spectra identifying neo N-terminal peptides from granzyme B cleavage sites in α4 integrin at (A) Asp110↓Asn101, (B) Asp166↓Met167, (C) Asp199↓Phe200, (D) Asp302↓Ser303, (E) Asp311↓Glu312, (F) Asp358↓Val359, (G) Asp482↓Arg483, (H) Asp488↓Val489, and (I) Glu856↓Gln857.

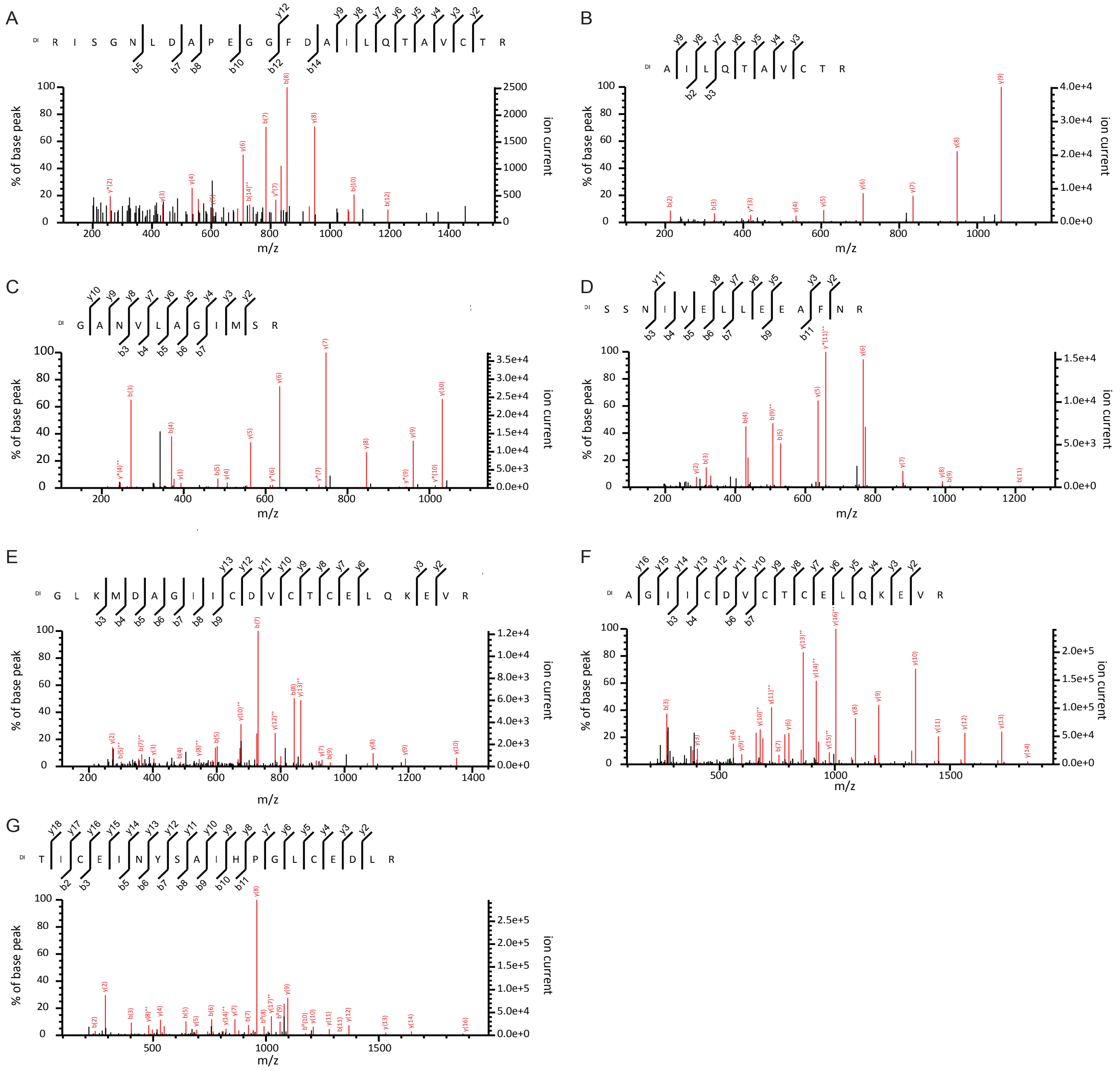

**Supplemental Figure S2:** Annotated MS/MS spectra identifying neo N-terminal peptides from granzyme B cleavage sites in β4 integrin at (A) Glu223↓Arg224, (B) Asp237↓Ala238, (C) Asp272↓Gly273, (D) Asp351↓Ser352, (E) Asp442↓Gly443, (F) Asp447↓Ala448, and (G) Asp611↓Ala612.

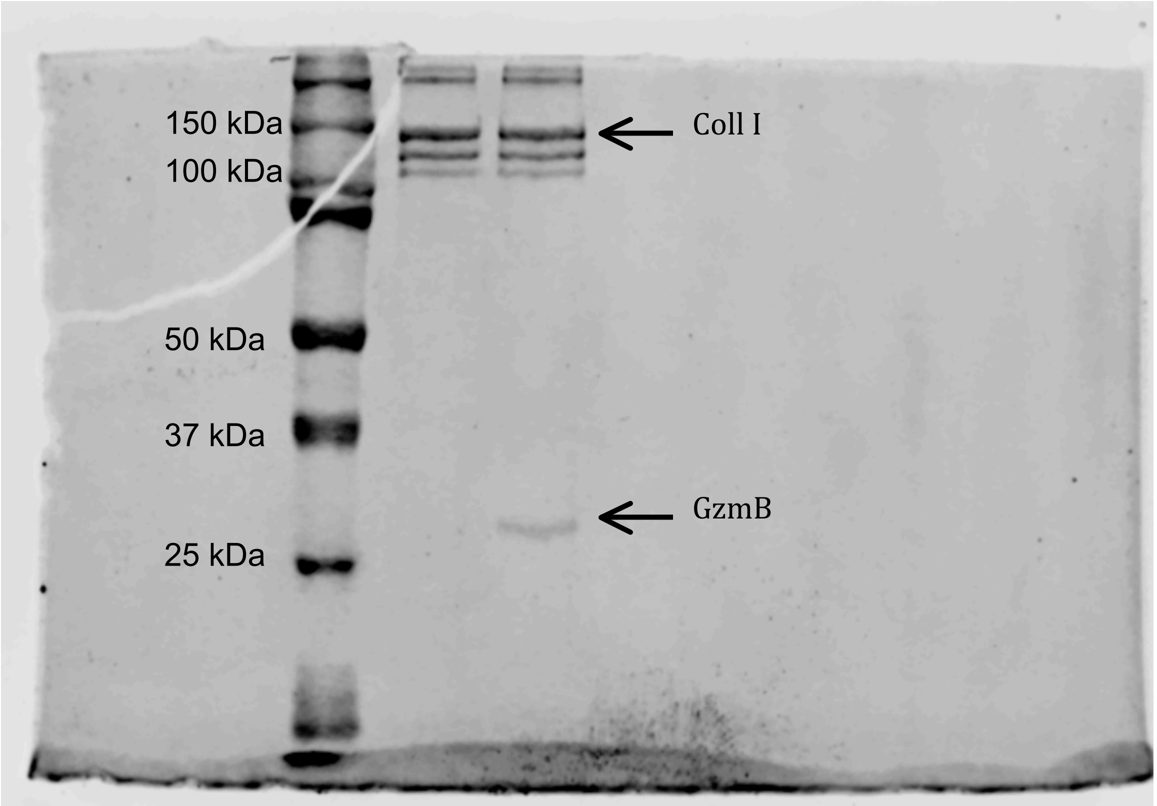


**Supplemental Figure S3:** 7.5% SDS-PAGE Coomassie staining of collagen I without and with the addition of 200 nM granzyme B. Black arrow indicates full length protein detected at 130 kDa, other bands represent collagen I splice variants and isoforms. Addition of granzyme B does not result in the appearance of cleavage bands. Coll I, collagen I; GzmB, granzyme B.

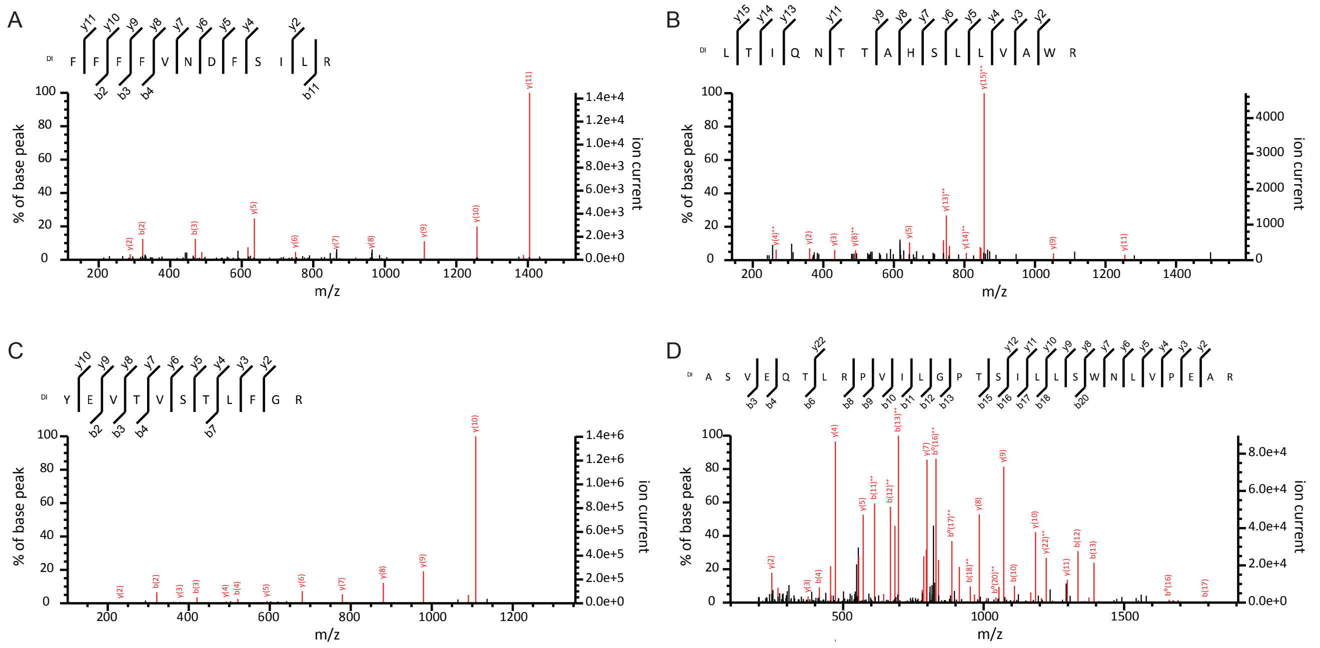


**Supplemental Figure S4:** Annotated MS/MS spectra identifying neo N-terminal peptides from granzyme B cleavage sites in collagen VII at (A) Asp193↓Phe194, (B) Glu332↓Leu333, (C) Asp390↓Tyr391, and (D) Asp414↓Ala415.

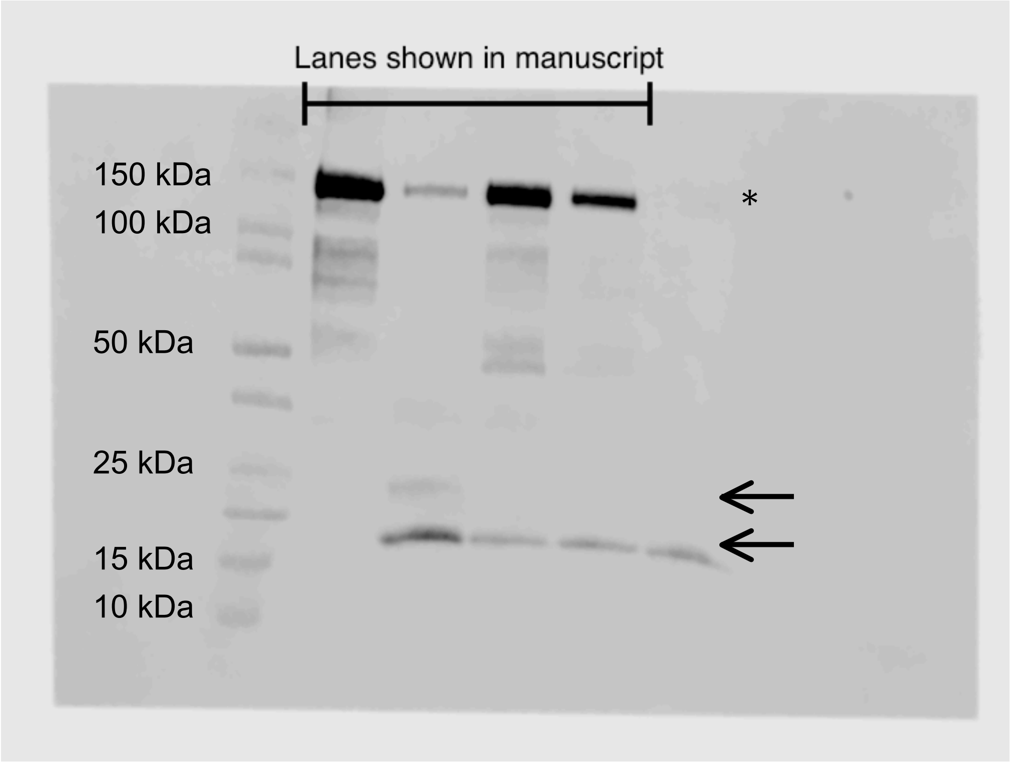


**Supplemental Figure S5:** Full-length blot for α6 integrin cleavage assay show in Figure 2: α6 integrin is a GzmB substrate and is reduced in sub-epidermal blistering. Black arrows indicate cleavage fragments and * indicates full-length protein.

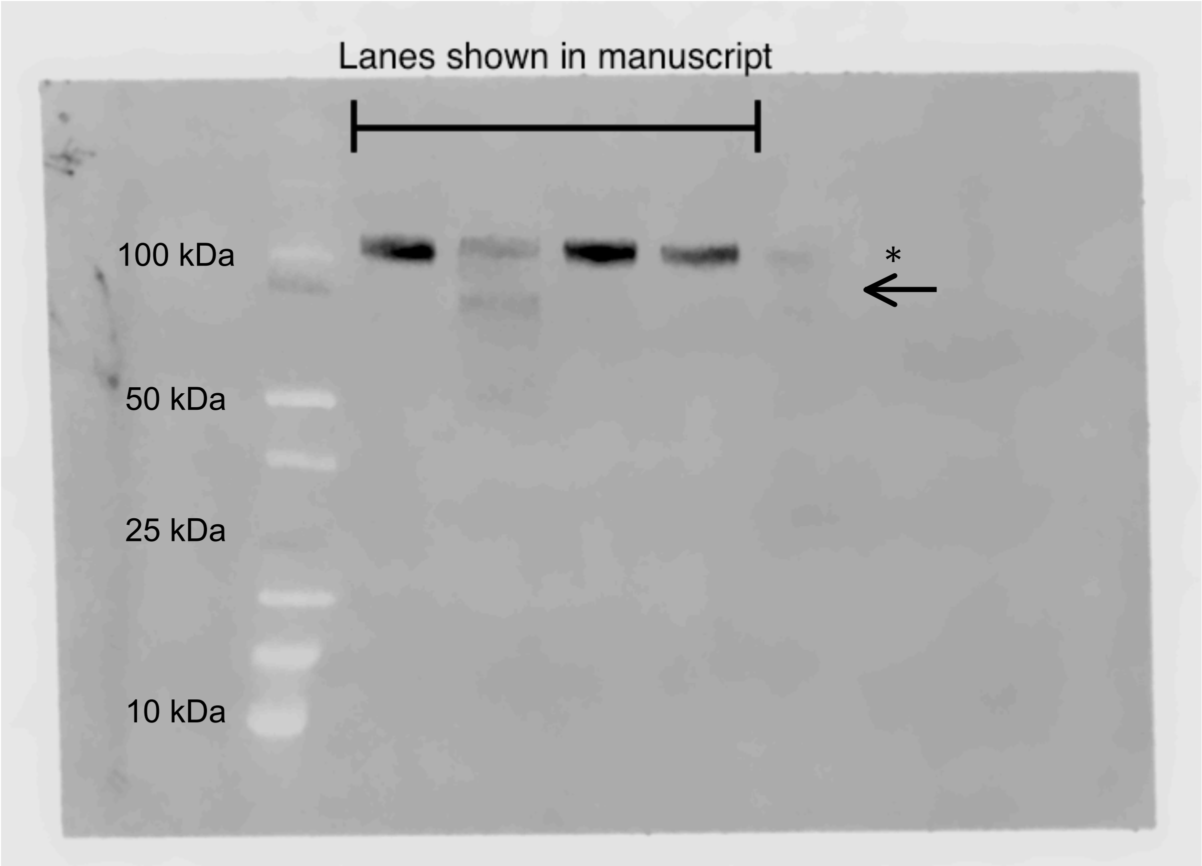


**Supplemental Figure S6:** Full-length blot for β4 integrin cleavage assay show in Figure 3: β4 integrin cleavage by GzmB and status in healthy skin versus and sub-epidermal blistering. Black arrow indicates cleavage fragment and * indicates full-length protein.

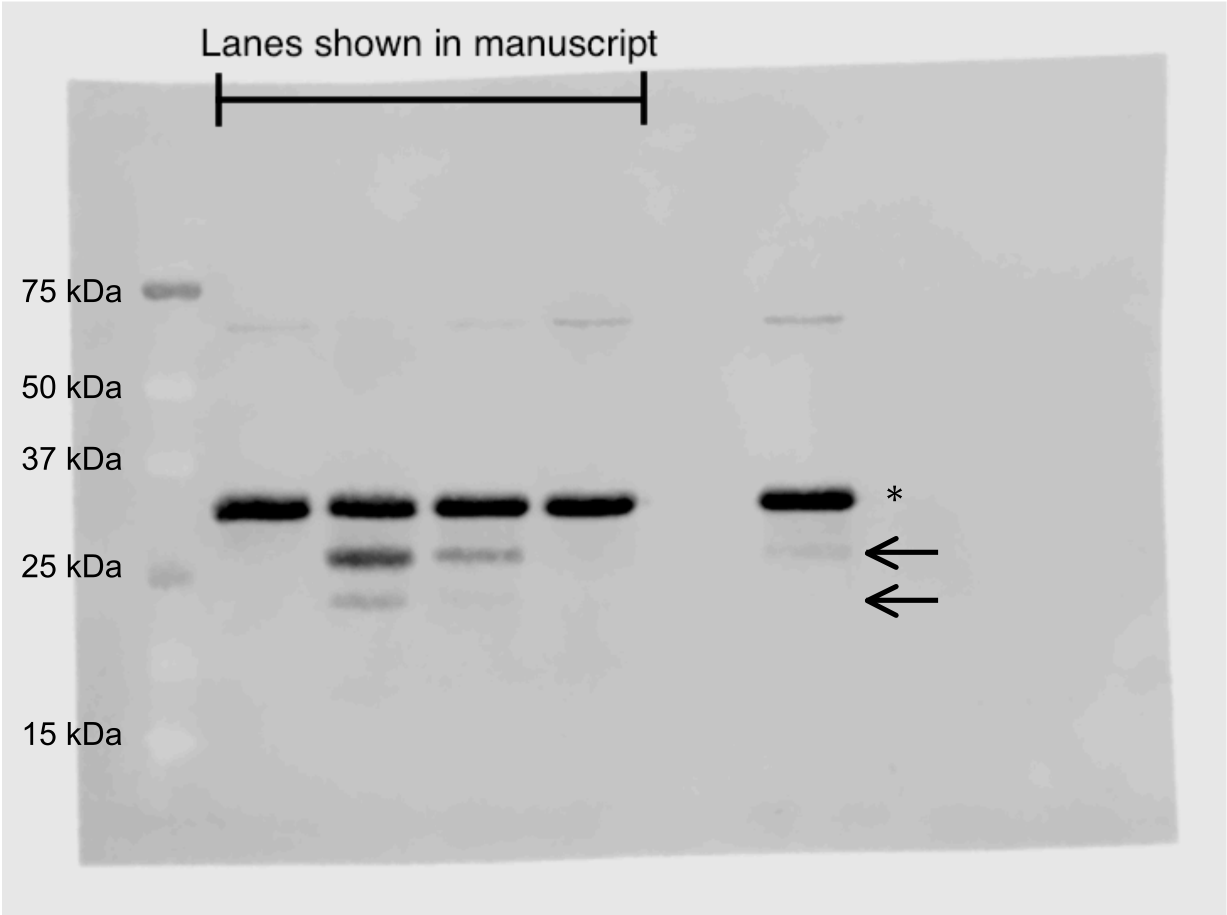


**Supplemental Figure S7:** Full-length blot for collagen VII cleavage assay show in Figure 4: GzmB-mediated collagen VII cleavage and histologic assessment in normal skin versus sub-epidermal blistering. Black arrows indicate cleavage fragments and * indicates full-length protein.

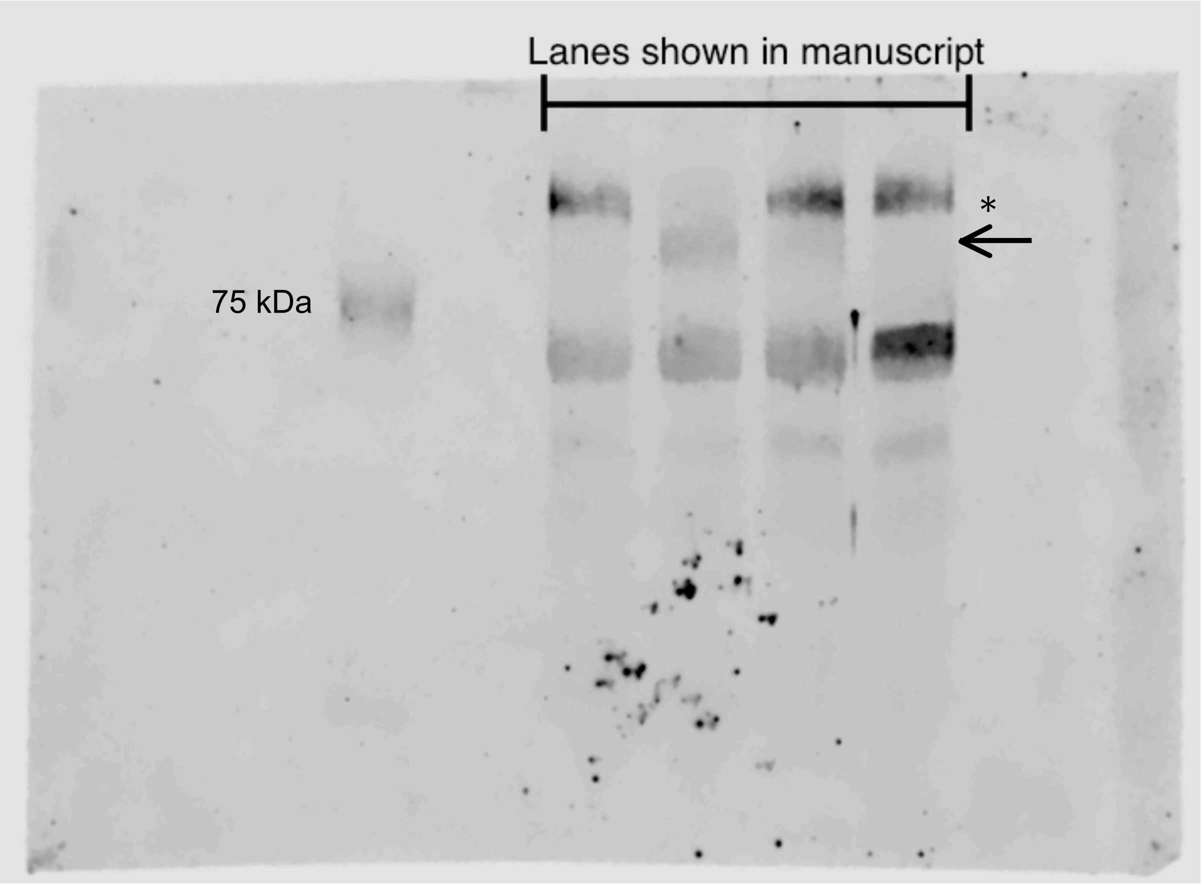


**Supplemental Figure S8:** Full-length blot for collagen XVII cleavage assay show in Figure 5: Collagen XVII is cleaved by GzmB and is absent in areas of epidermal separation in blistering skin biopsies. Black arrow indicates cleavage fragments and * indicates full-length protein.

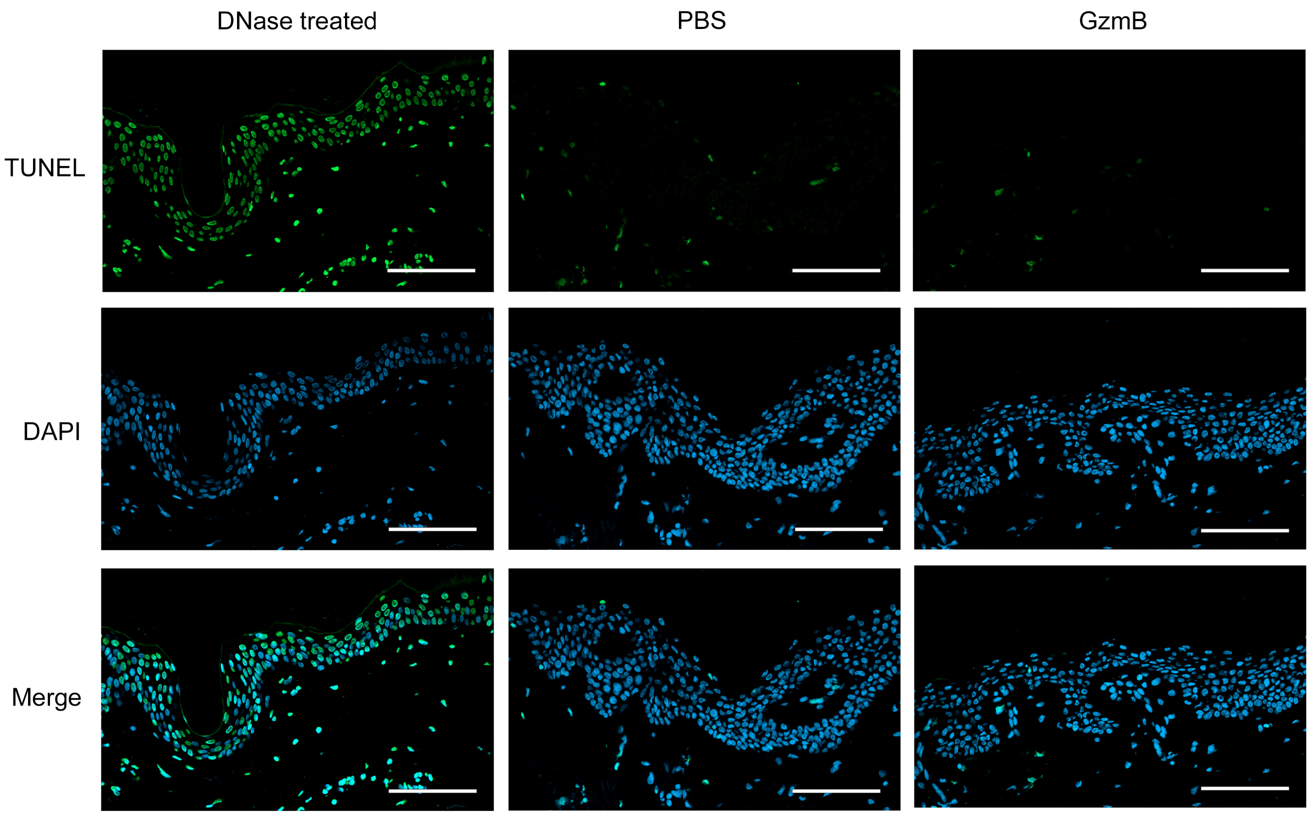


**Supplemental Figure S9:** *In situ* cell death detection assay (TUNEL) on skin incubated for 12 h at 37 °C in PBS and 200 nM GzmB. DNase treated sample was included as positive control. Cell death upon incubation in PBS and GzmB was minimal, and similar between the two conditions. Scale bars represent 100 µm.
